# Empyema Presentation Secondary to Streptococcus constellatus

**DOI:** 10.7759/cureus.43468

**Published:** 2023-08-14

**Authors:** Anji Li, Abrahim N Razzak, Milan R Patel, Pinky Jha, Abhijai Singh

**Affiliations:** 1 Medicine, Medical College of Wisconsin, Milwaukee, USA; 2 Internal Medicine, Medical College of Wisconsin, Milwaukee, USA

**Keywords:** immunocompetent individuals, acute hypoxemic respiratory failure, pleural empyema, streptococcus anginosus group, streptococcus constellatus

## Abstract

A 60-year-old male presented to our institution for abdominal pain and was later admitted to the intensive care unit for shock, acute hypoxemic respiratory failure, and acute kidney injury. He was subsequently found to have a large left-sided pleural effusion with empyema secondary to *Streptococcus constellatus*. With the emerging threat and growing prevalence of *Streptococcus anginosus* group pathogens, there is now greater clinical importance in identifying viridans streptococci at the species level. While immunosuppressed individuals are at greater risk of opportunistic pathogens, this case presentation demonstrated that *Streptococcus constellatus *can remain a serious community-acquired pathogen for the non-immunosuppressed. Continued interprofessional team care management and a greater look into the reasons for greater *Streptococcus anginosus* pathogenicity may be indicated.

## Introduction

*Streptococcus constellatus* is an opportunistic pathogen, previously thought of as a commensal organism, that belongs to the *Streptococcus anginosus* group, a viridans streptococcus [1]. This species is part of the normal oral flora, urogenital tract, and intestinal tract; however, as of late, they have been known to play an important role in purulent infection [1]. In this case presentation, we report a non-immunosuppressed patient with pleural empyema due to *S. constellatus*.

## Case presentation

**Figure 1:**
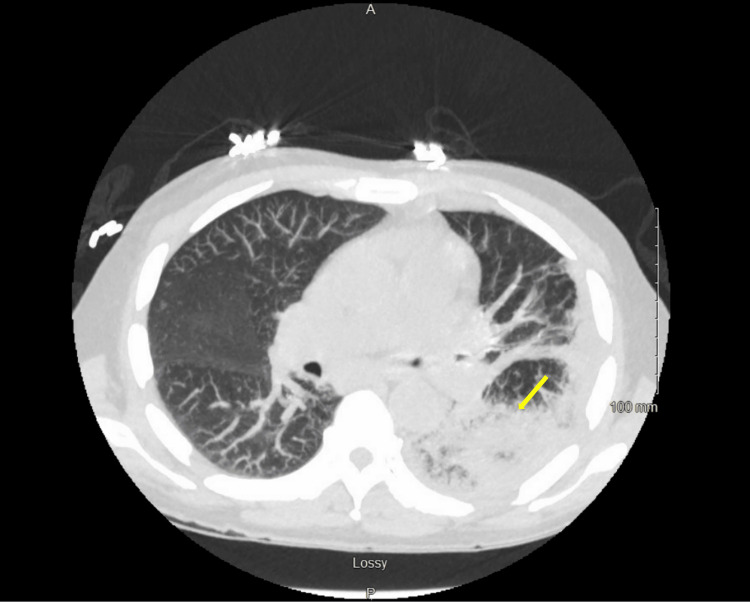
Computed tomography scan of the chest demonstrating near-complete consolidation of the left lower lobe and posterior left apical pleural effusion

A 60-year-old male with a past medical history of pancreatitis, alcohol use disorder, hypertension, gout, and hypothyroidism presented to the emergency department (ED) with epigastric abdominal pain that shoots to his back. He also reported a minimal oral intake over the last week, productive cough with green sputum, and significant drinking history (one shot/day). Upon arrival to the ED, he was tachycardic to 130 beats per minute, hypotensive to 98/60 mmHg, and hypoxemic with increased work of breathing, with an O_2_ saturation of 90%. Chest computed tomography (CT) showed near-complete consolidation of the left lower lobe (LLL) and posterior left apical pleural effusion (Figure 1). His serum creatinine was elevated at 4.77 mg/dL, and lactate level was elevated at 4.4 mmol/L on admission. He was started on empiric cefepime, azithromycin, and metronidazole and admitted to the medical intensive care unit for further management.

**Figure 2:**
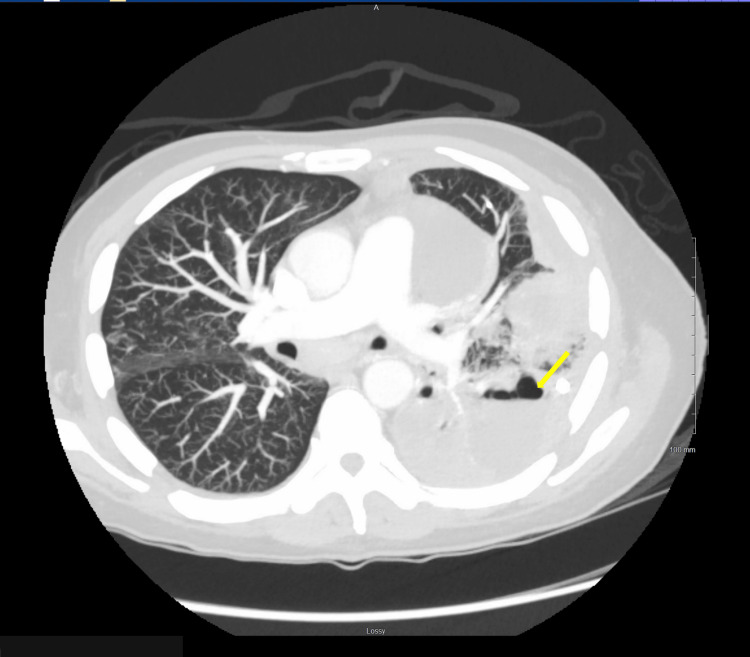
Computed tomography scan of the chest demonstrating the opacification and necrotization of the left lower lobe cavitary lesion

**Figure 3:**
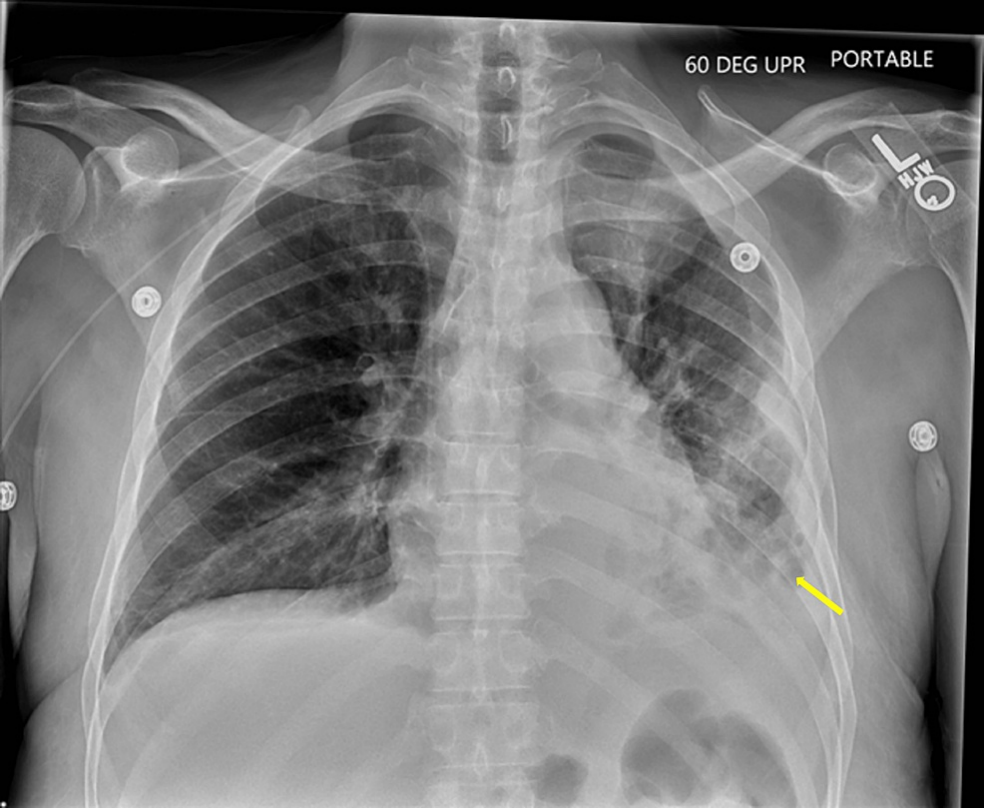
Chest X-ray demonstrating the stable left-sided pleural effusion relatively unchanged during admission

The patient's initial chest CT was concerning for a LLL pneumonia, most likely due to aspiration in the setting of nausea and chronic alcohol use. Infectious workup was ordered, and he was transitioned to intravenous (IV) piperacillin/tazobactam (Zosyn) with stress dose steroids. The patient underwent diagnostic thoracentesis of the left localized effusion two days post admission, with the removal of 700 cc purulent fluid. Fluid culture grew 4+ *S. constellatus*. Cytology was negative for malignant cells but showed numerous inflammatory cells consistent with empyema. Sputum culture grew normal oral flora. A repeat chest CT showed extensive opacification in the LLL with areas of cavitation, suggesting a necrotizing infection (Figure 2). Pulmonology was consulted for chest tube placement two days later. He also received two doses of tissue plasminogen activator (tPA) and dornase alfa, which were discontinued a day later when pleural fluid analysis showed hematocrit at 23%, raising concern for hemothorax. Leukocytosis persisted despite seven days of Zosyn, so he transitioned to cefazolin. Repeat studies eight days post admission noted of worsening left cavitary lesions that were not being fully evacuated by the chest tube. There were also progressive bilateral consolidations, concerning for necrotizing pneumonia. Infectious diseases transitioned the patient to a two-week course of intravenous cefepime and Flagyl. Cardiothoracic surgery and interventional radiology were consulted and decided to perform a second left apical chest tube placement with tPA 13 days post admission. Both chest tubes were removed four days after; both had minimal output. Chest X-ray showed the evidence of a stable effusion, and the patient was subsequently discharged after three days (Figure 3).

The patient was then monitored closely by his primary care providers and pulmonology specialists noting a steady normal recovery from the cavitary pneumonia and empyema.

## Discussion

In this case, *S. constellatus* played an aggressive pathogenic role in the development of pleural empyema. *Streptococcus constellatus* has been identified with empyema as far back as 1984 with increased numbers of cases being identified in recent years. While the organism displays greater virulence in immunosuppressed patients, *S. constellatus* empyema in multiple case reviews has been found more frequently in immunocompetent older males with comorbid diseases in nonhospital community settings. Common comorbidities reported in the literature include respiratory diseases, esophageal or gastric surgery, central nervous system infections, alcohol use disorder, hepatitis, pancreatitis, diabetes mellitus, and malignancy [2-4]. Previous cases have shown the utility of 16S ribosomal ribonucleic acid and next-generation sequencing in the identification of purulent fluid collected from similar cases [5,6], though in this case *S. constellatus* was identified through traditional culture means. There are many cases and reports that seem to indicate that there is a greater predilection for the infection to occur on the right side, which was thought to be due to greater aspiration risk in this demographic [7-9]. Empyema caused by *S. constellatus* was previously not taken seriously due to special culture conditions of oxygen/carbon dioxide the species required and was considered a commensal bacterium as opposed to a causal pathogen of infection. The association of empyema with *S. anginosus/viridans* was commonly overlooked in favor of the more common bacterial endocarditis with prior valvular damage. In recent years, there has been increasing evidence and awareness of a strong link between *S. constellatus* infection and right-sided pneumonia, pulmonary abscesses, acute bronchitis, or empyema [7-9].

*Streptococcus constellatus* infection is commonly polymicrobial, and a combination of organisms results in a generally worse clinical course due to the synergism of aerobes and anaerobes in the production of growth factors and more effective combined host immune evasion [10]. Acute solitary infection with *S. constellatus*, while severe, resulted in fatality in 10% of cases in animal models, which is similar to human case reports in which 1/15 patients died. The clinical course of the disease generally follows acute bronchopneumonia with the development of pulmonary edema within the first week and abscess/empyema formation within 1-2 weeks, which is generally when the patient presents to the hospital [6]. The treatment course of a typical patient in one case report was found to be antibiotic treatment for two weeks and chest tube drainage for 8.4 days, with total hospitalization time coming out to 19.6 days on average with a mortality rate of around 7% [2]. Complications associated with *S. constellatus* infection that have been reported in the past include pulmonic valve endocarditis, intracranial infection, and brain abscesses [11-13]. A common thread for successful recovery in severe case reports generally requires prompt chest tube placement or open drainage and irrigation [5,14]. Early surgical treatment in one study was associated with low mortality rate, shortened hospital course, and increase in number of patients who could be discharged home [6,15]. *Streptococcus​​​​​​*​ *constellatus* showed strong susceptibility to gentamicin, vancomycin, teicoplanin, and cefotaxime; moderate susceptibility to ciprofloxacin and penicillin; and greatest resistance to doxycycline, metronidazole, erythromycin, roxithromycin, and clindamycin, though local antibiotic susceptibility profiles should be kept in mind [16,17].

## Conclusions

In this case, *Streptococcus constellatus* played an aggressive role in pleural empyema for a non-immunosuppressed patient demonstrating the rise in *Streptococcus anginosus* group infections. Understanding that older male patients with comorbidities can have a greater possibility of infection with these organisms, timely identification of the organism and quick pus drainage/antibiotic usage can lead to better patient outcomes with this infection.
